# The causal associations of circulating amino acids with blood pressure: a Mendelian randomization study

**DOI:** 10.1186/s12916-022-02612-w

**Published:** 2022-10-28

**Authors:** Chenhao Lin, Zhonghan Sun, Zhendong Mei, Hailuan Zeng, Manying Zhao, Jianying Hu, Mingfeng Xia, Tao Huang, Chaolong Wang, Xin Gao, Yan Zheng

**Affiliations:** 1 State Key Laboratory of Genetic Engineering, Human Phenome Institute and School of Life Sciences, Fudan University, 2005 Songhu Road, Shanghai, 200433 China; 2 Ministry of Education Key Laboratory of Contemporary Anthropology, School of Life Sciences, Fudan University, Shanghai, China; 3 Department of Endocrinology and Metabolism, Zhongshan Hospital, Fudan Institute for Metabolic Diseases, and Human Phenome Institute, Fudan University, Shanghai, China; 4 Department of Epidemiology and Biostatistics, School of Public Health, Peking University, Beijing, China; 5 Department of Epidemiology and Biostatistics, School of Public Health, Tongji Medical College, Huazhong University of Science and Technology, Wuhan, Hubei China; 6 Ministry of Education Key Laboratory of Public Health Safety, School of Public Health, Fudan University, Shanghai, China

**Keywords:** Blood pressure, Hypertension, Amino acids, Metabolomics, Mendelian randomization

## Abstract

**Background:**

Circulating levels of amino acids were associated with blood pressure (BP) in observational studies. However, the causation of such associations has been hypothesized but is difficult to prove in human studies. Here, we aimed to use two-sample Mendelian randomization analyses to evaluate the potential causal associations of circulating levels of amino acids with BP and risk of hypertension.

**Methods:**

We generated genetic instruments for circulating levels of nine amino acids by conducting meta-analyses of genome-wide association study (GWAS) in UK Biobank participants with metabolomic data (*n* = 98,317) and another published metabolomics GWAS (*n* = 24,925). Data on the associations of the genetic variants with BP and hypertension were obtained in the UK Biobank participants without metabolomic data (*n* = 286,390). The causal effects were estimated using inverse-variance weighted method.

**Results:**

Significant evidence consistently supported the causal effects of increased branched-chain amino acids (BCAAs, i.e., leucine, isoleucine, and valine) levels on higher BP and risk of hypertension (all *P* < 0.006 after Bonferroni correction except for *P*_leucine-on-diastolicBP_ = 0.008). For example, per standard deviation higher of genetically predicted isoleucine levels were associated with 2.71 ± 0.78 mmHg higher systolic BP and 1.24 ± 0.34 mmHg higher diastolic BP, as well as with 7% higher risk of hypertension (odds ratio: 1.07, [95% CI: 1.04–1.10]). In addition, per standard deviation higher of genetically predicted glycine level was associated with lower systolic BP (− 0.70 ± 0.17 mmHg, *P* = 4.04 × 10^−5^) and a lower risk of hypertension (0.99 [0.98–0.99], *P* = 6.46 × 10^−5^). In the reverse direction, genetically predicted higher systolic BP was associated with lower circulating levels of glycine (− 0.025±0.008, *P* = 0.001).

**Conclusions:**

This study provides evidence for causal impacts of genetically predicted circulating BCAAs and glycine levels on BP. Meanwhile, genetically predicted higher BP was associated with lower glycine levels. Further investigations are warranted to clarify the underlying mechanisms.

**Supplementary Information:**

The online version contains supplementary material available at 10.1186/s12916-022-02612-w.

## Background

Cardiometabolic diseases remain the leading cause of morbidity and mortality globally, and high blood pressure (BP) is not only an endpoint but also a significant risk factor [1–3]. Although the application of biomarkers has augmented traditional cardiometabolic prediction and helped prevention, the aim of biomarker-guided prevention is still to be fully realized [4]. With the maturation of comprehensive metabolomic profiling technologies in the last decade, the pace of discoveries of disease-related biomarkers has been accelerated [5].

Identification of novel predictive biomarkers may help facilitate the detection and management of hypertension. To date, abundant evidence indicates that amino acids play a crucial role in cardiovascular physiology and pathology. Observational findings have suggested associations of circulating branched-chain amino acids (BCAAs) with cardiometabolic diseases and related risk factors [6–10], including hypertension [11, 12]. Increased dietary intake of aromatic amino acids, especially phenylalanine obtained from animal protein source, was associated with higher BP [13]. Other amino acids such as glycine, glutamine, histidine, and alanine also have been associated with hypertension [14–19]. Taken together, these pieces of observational evidence (Additional file 1: Table S1) have raised the hypotheses that these amino acids may causally contribute to the development of hypertension, and the other way around, higher BP levels may also cause a change in the circulating levels of amino acids. However, population-based intervention studies are lacking due to challenges such as cost and safety issues.

Mendelian randomization (MR) analysis has emerged as a valuable approach to estimate causal associations between exposures and outcomes [20]. In brief, MR uses single nucleotide polymorphisms (SNPs) from genome-wide association studies (GWASs) as proxies for exposure, namely instrumental variables (IVs), to make causal inferences. Based on the truth that the random allocation of parental alleles at conception, MR design is analogous to a randomized controlled trial and could therefore address conventional biases in observational studies, such as confounding and reverse causation. To date, the MR approach has contributed to several major inferences and validations of causal relationships between traditional risk factors and disease [20].

In the present study, we conducted two-sample MR analyses to infer the causality of circulating levels of amino acids with BP and risk of hypertension using individual-level data of European participants in UK Biobank and additional summary-level data of metabolomics GWAS by Kettunen et al. [21].

## Method

### Study population and design

**Fig. 1:**
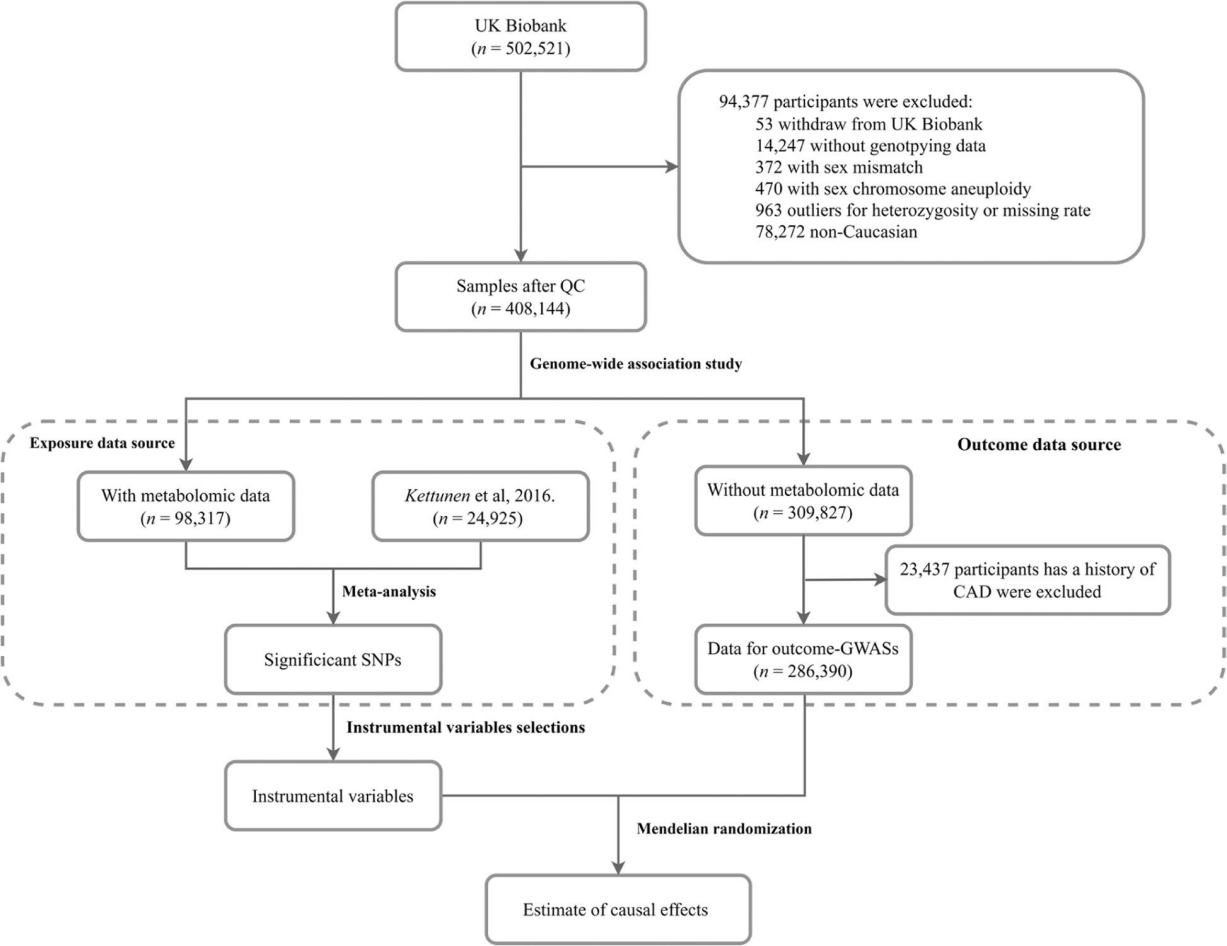
Flowchart of the analytical populations. Genetic variants for circulating levels of amino acids were calculated from a meta-analysis of GWAS using post-QC UK Biobank samples with metabolomics data (*n* = 98,317) and a published GWAS by Kettunen et al. (*n* = 24,925). Corresponding genetic effect sizes for these SNPs on BP traits and hypertension were calculated from the rest part of the UK Biobank population (*n* = 286,390). Causal effects of circulating levels of amino acids on BP and hypertension were estimated using a two-sample Mendelian randomization approach

The overview of the study is presented in Fig. 1. We used the individual-level data from UK Biobank, a large prospective cohort study of about 500,000 participants across the United Kingdom [22]. The detailed procedures of genotyping, imputation, and quality control of the genetic information in the UK Biobank have been described elsewhere [23]. Plasma metabolic biomarkers were measured in about 120,000 randomly selected participants using a high-throughput nuclear magnetic resonance (NMR)-based metabolite profiling platform developed by Nightingale Health Ltd (Helsinki, Finland). To date, the first release of metabolic data covers 249 metabolites, including nine amino acids. The unique data identifiers within the UK Biobank repository were shown in Additional file 1: Table S2.

In this study, we focused on the circulating amino acids reported to be associated with BP in observational studies [9–12, 14–19], including nine available in UK Biobank: leucine, isoleucine, valine, alanine, phenylalanine, tyrosine, histidine, glutamine, and glycine (Additional file 1: Table S2). Following central quality control procedures of UK Biobank [23], we excluded (1) individuals who had withdrawn consent, (2) non-Caucasian participants, (3) those without genotyping data, (4) those with sex chromosome aneuploidy, (5) those with a mismatch between genetically inferred sex and self-reported sex, and (6) outliers for heterozygosity or missing rate (Fig. 1) in the following analyses. We included two non-overlapping populations to conduct the two-sample MR analysis. One population included the individual-level data of 98,317 UK Biobank participants who had available metabolomics measurements together with another published summary-level data. The other population consisted of the remaining 309,827 UK Biobank participants without metabolomics data (Fig. 1). The protocol of the present study was approved by UK Biobank (#54294). This study followed the Strengthening the reporting of observational studies in epidemiology using Mendelian randomization (STROBE-MR, Additional file 2: STROBE-MR checklist) guideline [24].

### Data sources for exposures

We conducted GWAS analysis for circulating levels of amino acids in the UK Biobank samples of European ancestry with available metabolomic data (*n* = 98,317). We applied a rank-based inverse normal transformation to the circulating levels of amino acids. We used the Bayesian mixed-model association method [25, 26] allowing related individuals to perform GWAS in BOLT-LMM (2.3) software, with the adjustment of age, sex, fasting time, genotyping chips, assessment centers, and the top ten principal components.

For each amino acid, the results of GWAS in the UK Biobank were then meta-analyzed with the summary-level results from a published NMR-based metabolomics GWAS [21, 27] using METAL software [28]. Briefly, this external GWAS included 24,925 European participants from 14 cohorts whose blood metabolites were profiled by using quantitative NMR metabolomics platform. The covariates included age, sex, and fasting time [21]. Meta-analyses of these GWASs were conducted with genomic control correction under a fixed-effect model and the weights were based on standard error. We finally reached a sample size of 123,242 participants and restricted the current analyses to the 8,247,959 SNPs that were captured in both UK Biobank and this GWAS with a minor allele frequency > 1%.

To construct a valid IV, three basic assumptions must be satisfied: (1) the IV is associated with the exposure, (2) the IV is independent of any confounder in the association between the exposure and the outcome, and (3) the IV influences the outcome only through the exposure pathway. To meet assumption 1, we selected the corresponding SNPs for each circulating amino acid at the threshold of genome-wide significance (*P* < 5 × 10^−8^). Linkage disequilibrium was estimated between SNPs to select independent genetic variants using clump parameter in PLINK (1.9) software [29] (window size = 10,000 kb, *r*^2^ < 0.01 using 1000G Phase3 v5 EUR [GRCh37/hg19] as a reference panel). For those variants in linkage disequilibrium, we chose the one with the lowest *P*-value. We calculated the F statistic for each IV to assess the strength and avoid weak instrument bias [30]. As commonly used, the cut-off was set to *F* statistic > 10 [31]. The detailed information of the IV for each amino acid is shown in Additional file 1: Table S3. We excluded the SNPs that were associated with potential confounders (namely, coronary heart diseases, type 2 diabetes, and body mass index) at genome-wide significance threshold (*P* < 5 × 10^−8^) using available large-scale GWAS summary data from FinnGen study R5 release (https://www.finngen.fi/fi) [32] and GIANT consortium [33, 34] (Additional file 1: Table S4), respectively. The SNPs that were directly associated with outcomes were also excluded from analyses. In order to test the influence of the removal of SNPs on the results, we constructed broad sets of IVs that used all independent SNPs without any exclusion as a sensitivity analysis (Additional file 1: Table S5). All IVs in current analyses were robust instruments with F statistic >10 (Additional file 1: Tables S3 and S5).

Notably, several genetic variants for glycine are in (or near) carbamoyl-phosphate synthase 1 gene (*CPS1*, Additional file 1: Table S3). The *CPS1* gene exhibits broad effects on the circulating levels of multiple metabolites, such as trimethylamine N-oxide and its predecessors, which have been associated with cardiovascular events, including hypertension [35–38]. Due to the known pleiotropic effects of *CPS1* gene on human metabolism, we further generated one additional IV excluding variants in *CPS1* gene for glycine.

### Data sources for outcomes

Effect estimates for corresponding SNPs on systolic blood pressure (SBP), diastolic blood pressure (DBP), and hypertension were calculated in the UK Biobank participants without metabolomic data. Sitting BPs were measured twice using the Omron digital blood pressure monitor (Hoofddorp, Netherlands) or manual sphygmomanometer in the assessment center. Two automated and two manual BP measurements were averaged to derive SBP and DBP values for most participants, and the measured value was directly used for the participants who had single BP measurement only (0.084% of participants). Among the participants who reported taking BP-lowering medication, 15 and 10 mmHg were added to the measured SBP and DBP values, respectively [39]. We defined hypertension cases according to the usage of BP-lowering medication, the *International Classification of Diseases* (ICD-9 code 401 and ICD-10 code I10), or the average BP values of two measurements (average SBP>140 mmHg or average DBP > 90 mmHg). As many medications on coronary artery disease would affect the blood pressure levels, the participants who had a history of coronary artery disease or related procedures (Additional file 1: Table S2) were excluded in the association analysis of BP or hypertension, which is consistently with published GWASs of blood pressure and hypertension risk [40, 41].

The genetic estimates for BP traits and hypertension were calculated using linear mixed models with adjustment of age, sex, genotyping chips, assessment centers, and the top ten principal components in the BOLT-LMM (2.3) software [26].

### MR analysis

Data harmonization was conducted to make sure that the effect alleles were the same for both exposures and outcomes before every MR analysis. Because the summary statistics were calculated from the UK Biobank’s imputed genotype data, which were consistently aligned to the forward strand, we did not exclude palindromic SNPs with intermediate allele frequencies. For the primary analysis, we used the inverse variance weighted (IVW) method with multiplicative random effects [42], which is a powerful MR method under valid IVs and balanced pleiotropy assumptions, to estimate each circulating amino acid's causal effect on BP or risk of hypertension. Several sensitivity analyses were performed to assess the reliability of our findings, including MR-Egger regression [43], the median-based methods [44], and the mode-based method [45]. Specifically, under the weaker InSIDE (INstrument Strength Independent of Direct Effect) assumption, MR-Egger regression allows the IV to have horizontal pleiotropy if such pleiotropic effect is independent of the effect size of the genetic variant on the risk factor [43]. The directional pleiotropy can be estimated by conducting a hypothesis test for the intercept [46]. For the two median-based methods, the simple median method assumes that at least half of the variants are valid IVs, while weighted median method assumes that valid IVs provide more than half of the weight [44]. The mode-based method assumes a plurality of genetic variants is valid. Of note, these sensitivity analyses mentioned above are robust to violations of MR assumptions in some extent, but result in a relatively lower power to detect a causal effect.

Additionally, because circulating levels of glycine are correlated with that of BCAAs in human [47, 48], we performed multivariable MR through the IVW method to estimate the independent causal effect of circulating amino acids on outcomes. Because of the strong correlations among the BCAAs (Additional file 3: Fig. S1), we included each BCAA at one time in the multivariable MR model. The conditional F statistics [49] for each exposure was calculated to describe the instrument strength.

Results were presented as β ± SE mmHg change in BP measurements and odds ratios (ORs) (95% confidence intervals [95% CI]) for hypertension risk per one standard deviation (SD) increase in genetically predicted circulating amino acids level. A robust causal effect was defined by a consistency of directions across the above-mentioned MR methods. We used the MR-Egger method to detect the presence of potential pleiotropy. Heterogeneity was assessed using Cochran’s *Q* statistics for all circulating amino acids. If heterogeneity was detected, a multiplicative random-effects IVW method would be applied [50]. Meanwhile, a leave-one-out analysis was performed to check if any single SNP strongly drove the causal effects. To address the multiple testing issues, the threshold of statistical significance was defined as *P* < 0.006 (0.05/9 amino acids) after Bonferroni correction. Findings with 0.006 ≤ *P* < 0.05 were considered as associations of suggestively significant evidence. All the MR analyses were performed using R software (3.6.1) with the TwoSampleMR package (0.5.6) [51].

### Reverse MR analysis

To estimate the causal effects of genetically predicted BP or risk of hypertension on circulating levels of amino acids, we further performed bi-directional MR analyses using the IVW method. Independent genetic variants associated with the two BP traits and hypertension (*P* < 5 × 10^−8^; *r*^2^ < 0.01, European panel from the 1000 Genome Project) came from the GWASs in UK Biobank samples without metabolomic data as described above. The SNPs that were directly associated with circulating levels of amino acids were excluded from analyses. We constructed IVs including 360 SNPs for SBP, 358 for DBP, and 238 for hypertension, respectively (Additional file 1: Table S6). Data harmonization was also performed as described in forward MR analysis. The results were presented as β ± SE and the estimates represent SD change in circulating levels of amino acids per 10 mmHg of genetically predicted higher BP or per unit higher in log odds of hypertension risk. Again, MR-Egger regression, the median-based and mode-based methods were applied to test the reliability of the findings in sensitivity analyses. All the MR analyses were also performed using R software (3.6.1) with the TwoSampleMR package (0.5.6) [51].

## Results

### Summary of population characteristics

Among all included participants of UK Biobank (*n* = 384,707), the average age was 56.6 years, 44.4% were men, and 18.8% reported use of antihypertensive drugs (Additional file 1: Table S7). In the 286,390 participants without metabolomic data, the mean (SD) values of SBP and DBP were 138.02 (18.58) and 82.35 (10.06) mmHg, respectively, and 146 345 hypertensive participants were documented (Additional file 1: Table S7 and Additional file 3: Fig. S2). The distributions of circulating levels of amino acids are shown in Additional file 1: Table S7 and Additional file 3: Fig. S3.

### Association of genetically predicted circulating levels of amino acids with BP and risk of hypertension

**Fig. 2:**
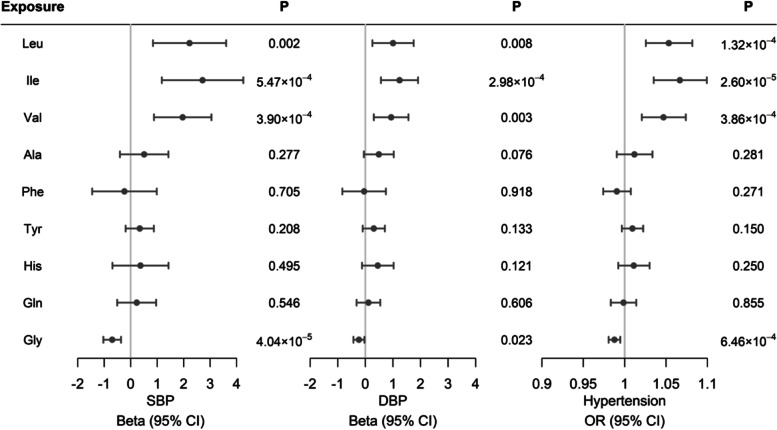
The evaluated causal effects of circulating levels of amino acids (per 1-SD increase) on systolic blood pressure (SBP), diastolic blood pressure (DBP), and risk of hypertension. Causal estimates are obtained using the inverse variance weighted method. Results are presented as mmHg change for BP traits and odds ratio for hypertension per one SD increase in genetically predicted circulating amino acids level. Leu, leucine; Ile, isoleucine; Val, valine; Ala, alanine; Phe, phenylalanine; Tyr, tyrosine; His, histidine; Gln, glutamine; Gly, glycine; OR, odds ratio and CI, confidence interval

Several potential causal effects of circulating amino acids on BP traits were observed (Fig. 2 and Additional file 1: Table S8). Genetically predicted higher levels of circulating BCAAs, leucine, isoleucine, and valine, were significantly associated with higher SBP (2.22 ± 0.70 mmHg per 1-SD increase in leucine; 2.71 ± 0.78 mmHg per 1-SD increase in isoleucine; 1.96 ± 0.55 mmHg per 1-SD increase in valine, all *P* < 0.006). Meanwhile, genetically predicted higher levels of isoleucine and valine were significantly associated with higher DBP (1.24 ± 0.34 mmHg per 1-SD increase in isoleucine; 0.94 ± 0.32 mmHg DBP per 1-SD increase in valine, all *P* < 0.006). We also observed suggestive associations of genetically predicted higher circulating leucine with higher DBP (1.01 ± 0.38 mmHg per 1-SD, *P* = 0.008). On the contrary, genetically predicted higher circulating glycine was associated with lower SBP (− 0.70 ± 0.17 mmHg per 1-SD, *P* = 4.04 × 10^−5^) and was suggestively associated with lower DBP (− 0.23 ± 0.10 mmHg per 1-SD, *P* = 0.02). Meanwhile, the causal effects of circulating levels of amino acids on the risk of hypertension were in accordance with the results from BP measurements. Genetically predicted higher circulating levels of three BCAAs were associated with a higher risk of hypertension (OR 1.05 [95% CI 1.03–1.08] per 1-SD increase in leucine, 1.07 [1.04-1.10] per 1-SD increase in isoleucine, 1.05 [1.02-1.07] per 1-SD increase in valine, all *P* < 0.006). Likewise, genetically predicted higher level of circulating glycine was associated with a lower risk of hypertension (0.99 [0.98–0.99], *P* < 0.006). Analyses using the broad sets of IVs showed consistent results, providing robust evidence on the causal effects of genetically determined circulating levels of BCAAs and glycine on BP (Additional file 1: Table S9 and Additional file 3: Fig. S4).

In addition, the association directions in sensitivity analyses using different methods were consistent with the results from the IVW method, and no evidence of pleiotropy was detected in the MR-Egger regression test (*P* > 0.05 for intercepts, Additional file 1: Table S8 and Additional file 3: Fig. S5). The leave-one-out analyses suggested that the associations were robust to exclusion of any single genetic variant (Additional file 3: Figs. S6-S8), though the Cochran’s *Q* test reported the presence of significant heterogeneity (Additional file 1: Table S8).

Because circulating levels of glycine are correlated with that of BCAAs in human [47, 48], we next conducted multivariable MR analyses, including glycine with one BCAA at a time as exposure to estimate their independent causal effects on BP traits and hypertension. After adjusting for isoleucine, the BCAA with the largest effect size, genetically predicted higher glycine was still significantly associated with a lower level of SBP, whereas the association with DBP was attenuated to null. Meanwhile, the positive association of the circulating level of isoleucine with BP traits and hypertension persisted (Additional file 1: Table S10). The results were similar when glycine was additionally adjusted for leucine or valine (Additional file 1: Table S10).

### Association of genetically predicted circulating glycine with BP and hypertension with respect to *CPS1* gene

**Fig. 3:**
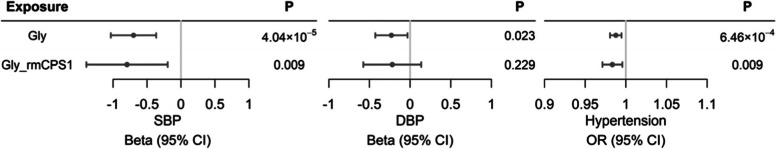
The evaluated causal effects of circulating levels of glycine (per 1-SD increase) on systolic blood pressure (SBP), diastolic blood pressure (DBP), and risk of hypertension using different genetic instrumental variables. Each row represents the raw glycine-related IV, including *CPS1* loci, and the IV excluding *CPS1* loci, respectively. Causal effects are estimated using the inverse-variance weighted method. Gly, glycine; Gly_rmCPS1, glycine-IV removing *CPS1* locus; OR, odds ratio and CI, confidence interval

In consideration of the remarkable pleiotropy of *CPS1* variants [36] and their strong effects on glycine (Additional file 1: Table S3), we investigated the associations of an additional IV for glycine without *CPS1* variants with outcomes. When using IV without the pleiotropic *CPS1* locus, only a suggestive association between circulating level of glycine and SBP (− 0.79 ± 0.30 mmHg per 1-SD, *P* = 0.009) or risk of hypertension (0.98 [0.97–0.99] per 1-SD, *P* = 0.009) were observed, and there was no causal effect of circulating glycine on DBP (*P* > 0.05, Fig. 3). The results were similar in sensitivity analyses, and the MR-Egger regression suggested that the results were not influenced by horizontal pleiotropy (all *P* > 0.05 for intercepts, Additional file 1: Table S8). Additionally, the combination of *CPS1* variants for glycine exhibited significant associations with lower BP levels and risk of hypertension (Additional file 1: Table S8).

### Reverse MR analysis assessing the causal effect of BP on circulating amino acids

We found that genetically predicted 10 mmHg higher in SBP was associated with a lower circulating level of glycine (− 0.025 ± 0.008, *P* = 0.001, Table 1). Genetically predicted higher BP or hypertension risk were not associated with other circulating levels of amino acids after Bonferroni correction (all *P*>0.006, Table 1 and Additional file 1: Table S11), though some suggestive associations were presented. For example, at suggestive significance level, genetically predicted higher SBP was positively associated with tyrosine, genetically predicted higher DBP was positively associated with isoleucine and valine but inversely associated with glycine, and genetically predicted higher risk of hypertension was associated with higher levels of circulating isoleucine and tyrosine, but lower levels of glycine (0.006 ≤ *P* < 0.05, Table 1 and Additional file 1: Table S11). In addition, although genetically predicted higher SBP was suggestively associated with histidine in the IVW method, the MR-Egger method found potential pleiotropy in the analysis with null causal effect (Additional file 1: Table S11). Besides, the directions were consistent in sensitivity analyses, and no other evidence of pleiotropy was detected in the MR-Egger regression test (Additional file 1: Table S11 and Additional file 3: Fig. S9). The Cochran’s *Q* test identified significant heterogeneity in the reverse MR analyses (Additional file 1: Table S11).

**Table 1:** The estimated causal effects of systolic blood pressure, diastolic blood pressure, and hypertension on circulating levels of amino acids

Outcome	Systolic blood pressure		Diastolic blood pressure		Hypertension	
	*β* ± SE	*P* value	*β* ± SE	*P* value	*β* ± SE	*P* value
Leu	0.010 ± 0.008	0.193	0.025 ± 0.014	0.071	0.066 ± 0.042	0.114
Ile	0.013 ± 0.008	0.099	0.030 ± 0.013	0.028	0.080 ± 0.040	0.048
Val	0.016 ± 0.008	0.054	0.029 ± 0.015	0.050	0.084 ± 0.044	0.058
Ala	0.001 ± 0.008	0.889	0.007 ± 0.013	0.589	− 0.0002 ± 0.040	0.995
Phe	0.001 ± 0.007	0.885	0.022 ± 0.013	0.093	0.039 ± 0.037	0.286
Tyr	0.020 ± 0.008	0.014	0.013 ± 0.015	0.404	0.124 ± 0.045	0.006
His	− 0.018 ± 0.007	0.011	− 0.02 ± 0.012	0.097	− 0.070 ± 0.037	0.060
Gln	− 0.018 ± 0.009	0.054	− 0.022 ± 0.016	0.151	− 0.053 ± 0.051	0.294
Gly	− 0.025 ± 0.008	0.001	− 0.036 ± 0.014	0.010	− 0.087 ± 0.041	0.036

Causal effects are estimated using the inverse variance weighted method

Results are presented as *β* ± SE. *β* means there is a *β* SD change in the circulating levels of amino acids per 10 mmHg of genetically predicted higher blood pressure or with the status of hypertension

*Abbreviations*: *Leu* leucine, *Ile* isoleucine, *Val* valine, *Ala* alanine, *Phe* phenylalanine, *Tyr* tyrosine, *His* histidine, *Gln* glutamine, *Gly* glycine, *SE* standard error

## Discussion

Using the two-sample MR approach, this study showed that genetically predicted higher circulating levels of BCAAs were associated with higher levels of BP and increased risk of hypertension. In contrast, genetically predicted higher circulating level of glycine was consistently associated with lower SBP and DBP, supporting a potential protective role in hypertension. Although the effect size was attenuated, genetically predicted glycine has a robust BP-lowering effect in the sensitivity analysis when pleiotropic *CPS1* variant were excluded. In the reverse MR analysis, we found that genetically predicted higher SBP was associated with a lower circulating level of glycine. Heterogeneity was detected among the instrumental variables. Though random-effects IVW method was applied and the results from the sensitivity analysis indicated the general robustness of our results, the biological interpretations should be made with caution.

The results from the present MR analysis are in line with previous observational studies, which suggested that high levels of circulating BCAAs were associated with higher BP measurements [52], or with an increased risk of incident hypertension [11]. Intriguingly, circulating levels of BCAAs are highly correlated and may have common genetic background. In the MR analyses, four common SNPs were identified as the shared IVs for leucine, isoleucine, and valine. The most significant variant, rs10014755 in *PPM1K* gene, encodes the mitochondrial phosphatase that regulate a rate-limiting enzyme for BCAA catabolism [8, 53], and two other variants, rs117643180 and rs2422358 in solute-carrier gene, affect amino acid transport [54]. These shared genetic mechanisms influence the transport and catabolism of BCAAs, thereby impact the circulating levels of BCAAs simultaneously.

One possible mechanism linking circulating BCAAs to BP involves the persistent activation of mTORC1 pathway in vascular system by BCAAs [8]. The activation of mTORC1 leads to changes of sympathetic nerve traffic [55], proliferation of vascular smooth muscle cell [56], kidney injury [57], and eventually the development of hypertension [58]. Another possible explanation is that higher levels of circulating BCAAs promote inflammation and oxidative stress in endothelial cells and elicit inflammatory cell adhesion and endothelial dysfunction, which contribute to increased risk of hypertension [59, 60]. Despite of these existing speculations, the investigation of the detailed mechanisms is still ongoing and further studies are warranted.

Furthermore, glycine is a non-essential amino acid that has been inversely associated with the risk of cardiovascular diseases [61] and type 2 diabetes [62, 63]. The potential protective role of glycine in hypertension prevention was supported by both cross-sectional studies [15] and prospective studies [14]. Of note, our results corroborated with previous observational findings and suggested a causal effect of genetically predicted higher circulating glycine on lower levels of BP or risk of hypertension. However, the original IV for glycine in the present study was largely driven by the *CPS1* locus, which was not only restricted to the glycine pathway but also exhibited significant effects on plasma trimethylamine N-oxide, betaine, and other intermediate metabolites in urea cycle [36]. A recent MR analysis, which aimed to evaluate the causal association between circulating glycine and coronary artery disease, also highlighted the potential pleiotropy of the *CPS1* rs1047891 SNP and did not obtain conclusive evidence for causality [64]. In addition, suggestive effects of glycine on SBP and risk of hypertension were still observed even when all *CPS1* variants were removed from the IV, and such findings were in line with another MR study based on six variants for glycine on cardiometabolic diseases when excluding the *CPS1* variant [65]. These consistent associations in analysis using IVs with and without *CPS1* variants suggested a robust and direct role of circulating glycine in BP management, independent of potential traditional risk factors.

While emerging observational studies implied the effects of circulating amino acids on hypertension risk [9], few studies explored whether hypertension would affect the circulating amino acids levels. In our reverse MR analyses, genetically predicted BP was associated with circulating levels of glycine and suggestively associated with circulating levels of isoleucine, tyrosine, and histidine. One possible explanation linking BP and glycine is that hypertension may reduce peripheral glucose utilization and promote insulin resistance and gluconeogenesis [66] and consequently accelerate the conversion of glycine to serine [67]. However, to our knowledge, there is no obvious mechanism well explaining the observed suggestive effects of higher BP on increased levels of BCAAs and aromatic amino acids. Further studies are needed to validate our observations and provide clues to the underlying mechanisms.

Our MR study supports a causal role of BCAAs in the pathophysiology of hypertension, and proposes a potential BP-lowering effect of glycine, implying the potential of clinical measurements and monitoring of circulating amino acids. This MR study has several strengths. First, to the best of our knowledge, this is the first study that has comprehensively scanned the causal effects of circulating amino acids on BP or hypertension using an MR approach. The two-sample MR design can minimize the bias inherent to observational study, such as residual confounding and reverse causality. Second, to calculate the genetic effect estimates for circulating amino acids, we performed meta-analyses using individual-level data in UK Biobank and summary statistics from another external GWAS, resulting in the largest sample size for the metabolomic GWAS to date. By splitting UK Biobank population, the current combination of data sources can avoid bias due to sample overlap and maintain a relatively large sample size with high SNP consistency. Meanwhile, the selection of SNPs was restricted by several procedures to meet basic assumptions for the MR approach and to ensure the validity and strength of IVs. Third, we conducted a thorough evaluation considering the pleiotropic effects of the *CPS1* variants, and our results further emphasized the robust roles of glycine in BP regulation. Finally, several sensitivity analyses were conducted to ensure consistency and robustness for our findings.

Of note, there are limitations in our study. First, due to the widespread pleiotropic effects of metabolite-related SNPs, it is hard to rule out the possibility that selected genetic variants may affect BP outcomes through an alternative causal pathway than through amino acid exposure. Nevertheless, we used several MR approaches as sensitivity analyses to ensure the robustness of our results against violation of MR assumptions. Another limitation is that the effect sizes of these amino acids on BP are relatively small. Finally, the present MR study relied on genetic estimates that derived from GWAS in participants of European descent, which though reduced population stratification, might limit the generalization of our findings to non-European populations.

## Conclusions

In summary, our findings support the causal association of higher circulating BCAAs levels and lower glycine levels with higher BP and elevated risk of hypertension. Furthermore, we are the first to report the associations of genetically predicted hypertension with circulating amino acids, suggesting an impact of hypertension on systematic metabolism. Our findings may improve the understanding of the connections between circulating levels of amino acids and BP. However, further studies are needed to clarify the potential role of amino acids in the pathophysiology of hypertension and identify plausible biological mechanisms.

## Supplementary Information

**Additional file 1: Table S1.** Overview of observational studies investigating associations of amino acids with blood pressure or hypertension. **Table S2.** Definitions and the unique data identifiers within UK Biobank data. **Table S3.** Characteristics of the instrumental variables for circulating levels of amino acids in primary analyses. **Table S4.** Association of selected SNPs in our primary analyses with potential confounders. **Table S5.** Characteristics of the broad sets of instrumental variables for circulating levels of amino acids in sensitivity analysis. **Table S6.** Characteristics of instrumental variables for SBP, DBP and hypertension in reverse MR analyses. **Table S7.** Characteristics of participants from UK Biobank included in the MR analyses. **Table S8.** Causal effects of circulating levels of amino acids on BP and hypertension using different methods. **Table S9.** Causal effects of circulating levels of amino acids on BP and hypertension using the broad sets of instrumental variables. **Table S10.** MVMR estimates the independent effects of BCAA and glycine on SBP, DBP and risk of hypertension. **Table S11.** Causal effects of BP and hypertension on circulating levels of amino acid using different methods.

**Additional file 2.** STROBE-MR checklist.

**Additional file 3: Figure S1.** Heatmap of Pearson correlation coefficients between circulating levels of amino acids in UK Biobank. **Figure S2.** Distribution of systolic blood pressure (SBP) and diastolic blood pressure (DBP) in 286 390 European participants without metabolomic data from UK Biobank. **Figure S3.** Distribution of circulating levels of amino acids in 98 317 European participants with metabolomic data from UK Biobank. **Figure S4.** Estimated causal effects of circulating levels of amino acids on blood pressure and risk of hypertension. **Figure S5.** Scatter plots of SNPs used as instrumental variables for the MR analyses of circulating amino acids with (A) systolic blood pressure, (B) diastolic blood pressure and (C)hypertension. **Figure S6.** Leave-one-out plots to assess if a single SNP is driving the causal effects of circulating amino acids on systolic blood pressure (SBP). **Figure S7.** Leave-one-out plots to assess if a single SNP is driving the causal effects of circulating amino acids on diastolic blood pressure (DBP). **Figure S8.** Leave-one-out plots to assess if a single SNP is driving the causal effects of circulating amino acids on hypertension. **Figure S9.** Scatter plots of SNPs used as IVs for the reverse MR analyses of (A) systolic blood pressure, (B) diastolic blood pressure and (C) hypertension with circulating levels of amino acids.

## Data Availability

The UK Biobank data are available from the UK Biobank on application (www.ukbiobank.ac.uk/). All the supporting data for MR analyses are available within the article and supporting information. Further data can be obtained by a reasonable request to the corresponding author.

## Abbreviations

BCAA: Branched-chain amino acid
BP: Blood pressure
CI: Confidence interval
*CPS1*: Carbamoyl-phosphate synthase 1
DBP: Diastolic blood pressure
GWAS: Genome-wide association study
ICD: International Classification of Diseases
IV: Instrumental variable
IVW: Inverse variance weighted
MR: Mendelian randomization
NMR: Nuclear magnetic resonance
OR: Odds ratio
SBP: Systolic blood pressure
SD: Standard deviation
SNP: Single nucleotide polymorphism
STROBE-MR: STrengthening the Reporting of Observational Studies in Epidemiology using Mendelian Randomisation
