# Supplementary material for: The causal associations of circulating amino acids with blood pressure: a Mendelian randomization study
Source: BMC Med. 2022 Oct 28;20:414. doi: 10.1186/s12916-022-02612-w (PMC9615211; doi:10.1186/s12916-022-02612-w)
Supplement: Supplementary file 2 — Additional file 2. STROBE-MR checklist. [file 12916_2022_2612_MOESM2_ESM.docx]

STROBE-MR checklist of recommended items to address in reports of Mendelian randomization studies1 2

| **Item No.** | **Section** | **Checklist item** | **Page No.** | Relevant text from manuscript |
| --- | --- | --- | --- | --- |
| 1 | **TITLE and ABSTRACT** | Indicate Mendelian randomization (MR) as the study’s design in the title and/or the abstract if that is a main purpose of the study | 1-3 | The term "Mendelian randomization" was included both in the title and the abstract. |
| **INTRODUCTION** | | | | |
| 2 | **Background** | Explain the scientific background and rationale for the reported study. Is causality between exposure and outcome plausible? Justify why MR is a helpful method to address the study question. | 4,5 | Introduction, Paragraph 1-3 |
| 3 | **Objectives** | State specific objectives clearly, including pre-specified causal hypotheses (if any). State that MR is a method that, under specific assumptions, intends to estimate causal effects | 4,5 | Introduction, Paragraph 3, 4 |
| **METHODS** | | | | |
| 4 | **Study design and data sources** | Present key elements of the study design early in the article. Consider including a table listing sources of data for all phases of the study. For each data source contributing to the analysis, describe the following: |  |  |
|  | a) | Setting: Describe the study design and the underlying population, if possible. Describe the setting, locations, and relevant dates, including periods of recruitment, exposure, follow-up, and data collection, when available. | 5,6 | Method, Section “Study population and design". The flowchart of this study was also shown in Figure 1. |
|  | b) | Participants: Give the eligibility criteria, and the sources and methods of selection of participants. Report the sample size, and whether any power or sample size calculations were carried out prior to the main analysis | 6-9 | Information about the GWAS studies was provided in the Method, Sections “Data sources for exposures" and "Data sources for outcomes". |
|  | c) | Describe measurement, quality control and selection of genetic variants | 6,7 | Method, Section “Data sources for exposures", Paragraph 3,4 |
|  | d) | For each exposure, outcome, and other relevant variables, describe methods of assessment and diagnostic criteria for diseases | 5-9 | The information of metabolite profiling and blood pressure measurements in UK Biobank was described in Method, Section "Study population and design". The criteria for hypertension was described in Method, Section "Data sources for outcomes". |
|  | e) | Provide details of ethics committee approval and participant informed consent, if relevant | 5 | Method, Section “Study population and design”, Paragraph 1-2 |
| S | **Assumptions** | Explicitly state the three core IV assumptions for the main analysis (relevance, independence and exclusion restriction) as well assumptions for any additional or sensitivity analysis | 7-10 | Method, Section “Data sources for exposures", Paragraph 3,4  Method, Section “MR Analysis", Paragraph 1 |
| 6 | **Statistical methods: main analysis** | Describe statistical methods and statistics used |  |  |
|  | a) | Describe how quantitative variables were handled in the analyses (i.e., scale, units, model) | 6-8 | Method, Section “Data sources for exposures", Paragraph 1,2 Method, Section “Data sources for outcomes", Paragraph 1 |
|  | b) | Describe how genetic variants were handled in the analyses and, if applicable, how their weights were selected | 9,10 | Method, Section “MR Analysis", Paragraph 1 |
|  | c) | Describe the MR estimator (e.g. two-stage least squares, Wald ratio) and related statistics. Detail the included covariates and, in case of two-sample MR, whether the same covariate set was used for adjustment in the two samples | 10,11 | Method, Section “MR Analysis", Paragraph 3 |
|  | d) | Explain how missing data were addressed | - | Not applicable to our study |
|  | e) | If applicable, indicate how multiple testing was addressed | 10,11 | Method, Section “MR Analysis", Paragraph 3 |
| 7 | **Assessment of assumptions** | Describe any methods or prior knowledge used to assess the assumptions or justify their validity | 7,8 | Method, Section "Data sources for exposures", Paragraph 3,4 |
| 8 | **Sensitivity analyses and additional analyses** | Describe any sensitivity analyses or additional analyses performed (e.g. comparison of effect estimates from different approaches, independent replication, bias analytic techniques, validation of instruments, simulations) | 9, 10 | Method, Section “MR Analysis", Paragraph 1 |
| 9 | **Software and pre registration** |  |  |  |
|  | a) | Name statistical software and package(s), including version and settings used | 10,11 | Method, Section “MR Analysis", Paragraph 3 |
|  | b) | State whether the study protocol and details were pre-registered (as well as when and where) | 6 | Method, Section "Study population and design ", Paragraph 2 |
|  |  | **RESULTS** |  |  |
| 10 | **Descriptive data** |  |  |  |
|  | a) | Report the numbers of individuals at each stage of included studies and reasons for exclusion. Consider use of a flow diagram | 12 | Result, Section "Summary of population characteristics". The flowchart of this study was also shown in Figure 1. |
|  | b) | Report summary statistics for phenotypic exposure(s), outcome(s), and other relevant variables (e.g. means, SDs, proportions) | 12 | Result, Section "Summary of population characteristics". Detailed summary statistics was also shown in Supplementary Table 8, Supplementary Figure 2 and 3. |
|  | c) | If the data sources include meta-analyses of previous studies, provide the assessments of heterogeneity across these studies | - | Not applicable to our study |
|  | d) | For two-sample MR: |  |  |
|  |  | i. Provide justification of the similarity of the genetic variant-exposure associations between the exposure and outcome samples | - | Not applicable to our study |
|  |  | ii. Provide information on the number of individuals who overlap between the exposure and outcome studies | 6-7 | There was no overlapping individual between the exposure and outcome studies, which was described in Method, “Study population and design”, Paragraph 2. |
| 11 | **Main results** |  |  |  |
|  | a) | Report the associations between genetic variant and exposure, and between genetic variant and outcome, preferably on an interpretable scale | 6-9 | Method, Sections “Data sources for exposures" and “Data sources for outcomes". Summary statistics was also shown in Supplementary Table 3 |
|  | b) | Report MR estimates of the relationship between exposure and outcome, and the measures of uncertainty from the MR analysis, on an interpretable scale, such as odds ratio or relative risk per SD difference | 12-15 | Our results were given in terms of beta ± SE for BP measurements and odds ratios (95% confidence intervals) for risk of hypertension throughout the results section. Detailed information was shown in Figure 2-3, Table 1 and Supplementary Table 8-11. |
|  | c) | If relevant, consider translating estimates of relative risk into absolute risk for a meaningful time period | - | Not applicable to our study |
|  | d) | Consider plots to visualize results (e.g. forest plot, scatterplot of associations between genetic variants and outcome versus between genetic variants and exposure) | 32,33 | Forest plots was shown in Figure 2 and 3. Scatterplots of genetic variants were shown in Supplementary Figure. |
| 12 | **Assessment of assumptions** |  |  |  |
|  | a) | Report the assessment of the validity of the assumptions | 12 | We assessed the validity using several sensitivity analyses, which was describe in the “Association of genetically predicted circulating levels of amino acids with BP and risk of hypertension” section of results section. We also calculated the F statistic for each IV in Supplementary Table 3. |
|  | b) | Report any additional statistics (e.g., assessments of heterogeneity across genetic variants, such as *I2*, Q statistic or E-value) | - | Additional statistics were presented in Supplementary Table 8-11. |
| 13 | **Sensitivity analyses and additional analyses** |  |  |  |
|  | a) | Report any sensitivity analyses to assess the robustness of the main results to violations of the assumptions | 12,13 | Result, Section " Association of genetically predicted circulating levels of amino acids with BP and risk of hypertension ", Paragraph 2 and 3. |
|  | b) | Report results from other sensitivity analyses or additional analyses | 12,13 | Result, Section " Association of genetically predicted circulating levels of amino acids with BP and risk of hypertension ", Paragraph 2 and 3. Supplementary Table 8-11 |
|  | c) | Report any assessment of direction of causal relationship (e.g., bidirectional MR) | 14,15 | Result, Section "Reverse MR analysis assessing the causal effect of BP on circulating amino acids" |
|  | d) | When relevant, report and compare with estimates from non-MR analyses |  | Not applicable to our study |
|  | e) | Consider additional plots to visualize results (e.g., leave-one-out analyses) | - | Additional plots were presented in Supplementary Figure 4-9 |
|  |  | **DISCUSSION** |  |  |
| 14 | **Key results** | Summarize key results with reference to study objectives | 15 | Discussion, Paragraph 1 |
| 15 | **Limitations** | Discuss limitations of the study, taking into account the validity of the IV assumptions, other sources of potential bias, and imprecision. Discuss both direction and magnitude of any potential bias and any efforts to address them | 18,19 | We discussed the limitation of our study in Discussion, Paragraph 7. |
| 16 | **Interpretation** |  |  |  |
|  | a) | Meaning: Give a cautious overall interpretation of results in the context of their limitations and in comparison with other studies | 15-17 | Discussion, Paragraph 2-5 |
|  | b) | Mechanism: Discuss underlying biological mechanisms that could drive a potential causal relationship between the investigated exposure and the outcome, and whether the gene-environment equivalence assumption is reasonable. Use causal language carefully, clarifying that IV estimates may provide causal effects only under certain assumptions | 15-17 | Discussion, Paragraph 3-5 |
|  | c) | Clinical relevance: Discuss whether the results have clinical or public policy relevance, and to what extent they inform effect sizes of possible interventions | 17 | Discussion, Paragraph 6 |
| 17 | **Generalizability** | Discuss the generalizability of the study results (a) to other populations, (b) across other exposure periods/timings, and (c) across other levels of exposure | 18 | Discussion, Paragraph 7 |
|  |  | **OTHER INFORMATION** |  |  |
| 18 | **Funding** | Describe sources of funding and the role of funders in the present study and, if applicable, sources of funding for the databases and original study or studies on which the present study is based | 20 | Section “Funding”. |
| 19 | **Data and data sharing** | Provide the data used to perform all analyses or report where and how the data can be accessed, and reference these sources in the article. Provide the statistical code needed to reproduce the results in the article, or report whether the code is publicly accessible and if so, where | 20 | Section “Availability of data and materials” |
| 20 | **Conflicts of Interest** | All authors should declare all potential conflicts of interest | 20 | Section “Competing interests” |

**1. Skrivankova VW, Richmond RC, Woolf BAR, Yarmolinsky J, Davies NM, Swanson SA, et al. Strengthening the Reporting of Observational Studies in Epidemiology using Mendelian Randomization (STROBE-MR) Statement. JAMA. 2021;under review.**

**2. Skrivankova VW, Richmond RC, Woolf BAR, Davies NM, Swanson SA, VanderWeele TJ, et al. Strengthening the Reporting of Observational Studies in Epidemiology using Mendelian Randomisation (STROBE-MR): Explanation and Elaboration. BMJ. 2021;375:n2233.**
