# Supplementary material for: The causal associations of circulating amino acids with blood pressure: a Mendelian randomization study
Source: BMC Med. 2022 Oct 28;20:414. doi: 10.1186/s12916-022-02612-w (PMC9615211; doi:10.1186/s12916-022-02612-w)
Supplement: Supplementary file 3 — Additional file 3: Figure S1. Heatmap of Pearson correlation coefficients between circulating levels of amino acids in UK Biobank. Figure S2. Distribution of systolic blood pressure (SBP) and diastolic blood pressure (DBP) in 286 390 European participants without metabolomic data from UK Biobank. Figure S3. Distribution of circulating levels of amino acids in 98 317 European participants with metabolomic data from UK Biobank. Figure S4. Estimated causal effects of circulating levels of amino acids on blood pressure and risk of hypertension. Figure S5. Scatter plots of SNPs used as instrumental variables for the MR analyses of circulating amino acids with (A) systolic blood pressure, (B) diastolic blood pressure and (C)hypertension. Figure S6. Leave-one-out plots to assess if a single SNP is driving the causal effects of circulating amino acids on systolic blood pressure (SBP). Figure S7. Leave-one-out plots to assess if a single SNP is driving the causal effects of circulating amino acids on diastolic blood pressure (DBP). Figure S8. Leave-one-out plots to assess if a single SNP is driving the causal effects of circulating amino acids on hypertension. Figure S9. Scatter plots of SNPs used as IVs for the reverse MR analyses of (A) systolic blood pressure, (B) diastolic blood pressure and (C) hypertension with circulating levels of amino acids. [file 12916_2022_2612_MOESM3_ESM.docx]

**SUPPLEMENTARY FIGURES**

**The causal associations of circulating amino acids with blood pressure: a Mendelian randomization study**

Chenhao Lin^1,2#^, Zhonghan Sun^1#^, Zhendong Mei^1#^, Hailuan Zeng^3^, Manying Zhao^1^, Jianying Hu^1^, Mingfeng Xia^3^, Tao Huang^4^, Chaolong Wang^5^, Xin Gao^3^, Yan Zheng^1, 6*^

^1^ State Key Laboratory of Genetic Engineering, Human Phenome Institute, School of Life Sciences, Fudan University, Shanghai, China

^2^ Ministry of Education Key Laboratory of Contemporary Anthropology, School of Life Sciences, Fudan University, Shanghai, China

^3^ Department of Endocrinology and Metabolism, Zhongshan Hospital, Fudan Institute for Metabolic Diseases, and Human Phenome Institute, Fudan University, Shanghai, China

^4^ Department of Epidemiology and Biostatistics, School of Public Health, Peking University, Beijing, China

^5^ Department of Epidemiology and Biostatistics, School of Public Health, Tongji Medical College, Huazhong University of Science and Technology, Wuhan, Hubei, China

^6^ Ministry of Education Key Laboratory of Public Health Safety, School of Public Health, Fudan University, Shanghai, China.

#These authors contributed equally to this work.

*Correspondences to Yan Zheng, State Key Laboratory of Genetic Engineering, Human Phenome Institute, and School of Life Sciences, Fudan University, 2005 Songhu Road, Shanghai, China 200433, Tel: +86-21-31246764, Email: [yan_zheng@fudan.edu.cn](mailto:yan_zheng@fudan.edu.cn)


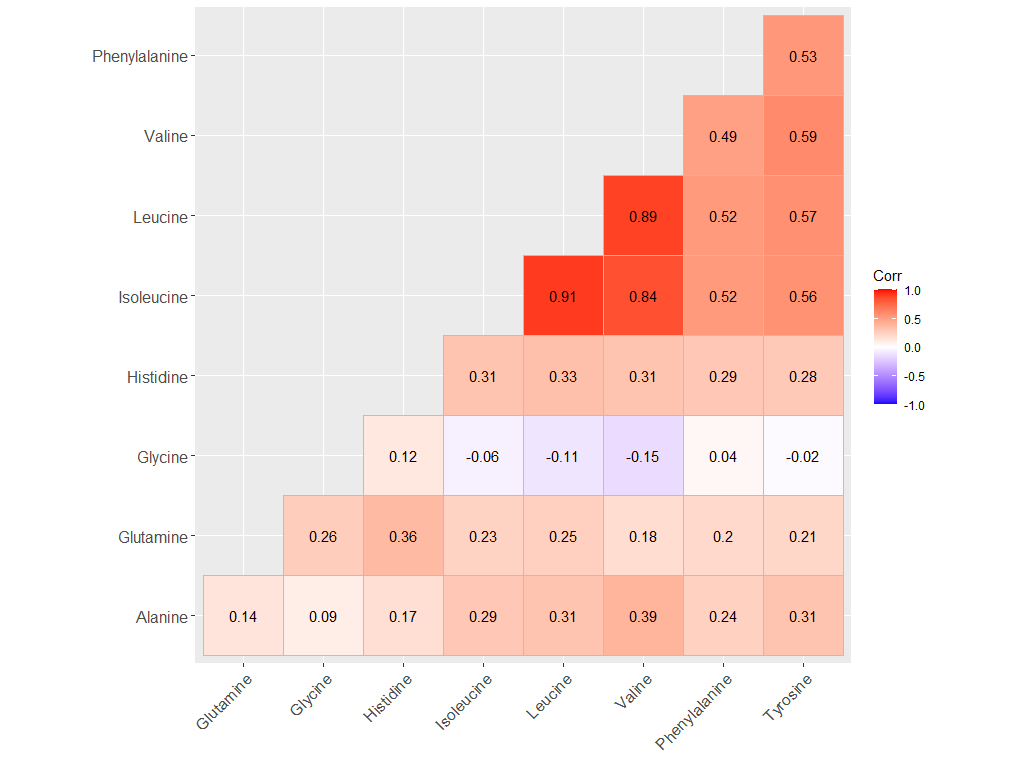


**Fig. S1 Heatmap of Pearson correlation coefficients between circulating levels of amino acids in UK Biobank.**

**
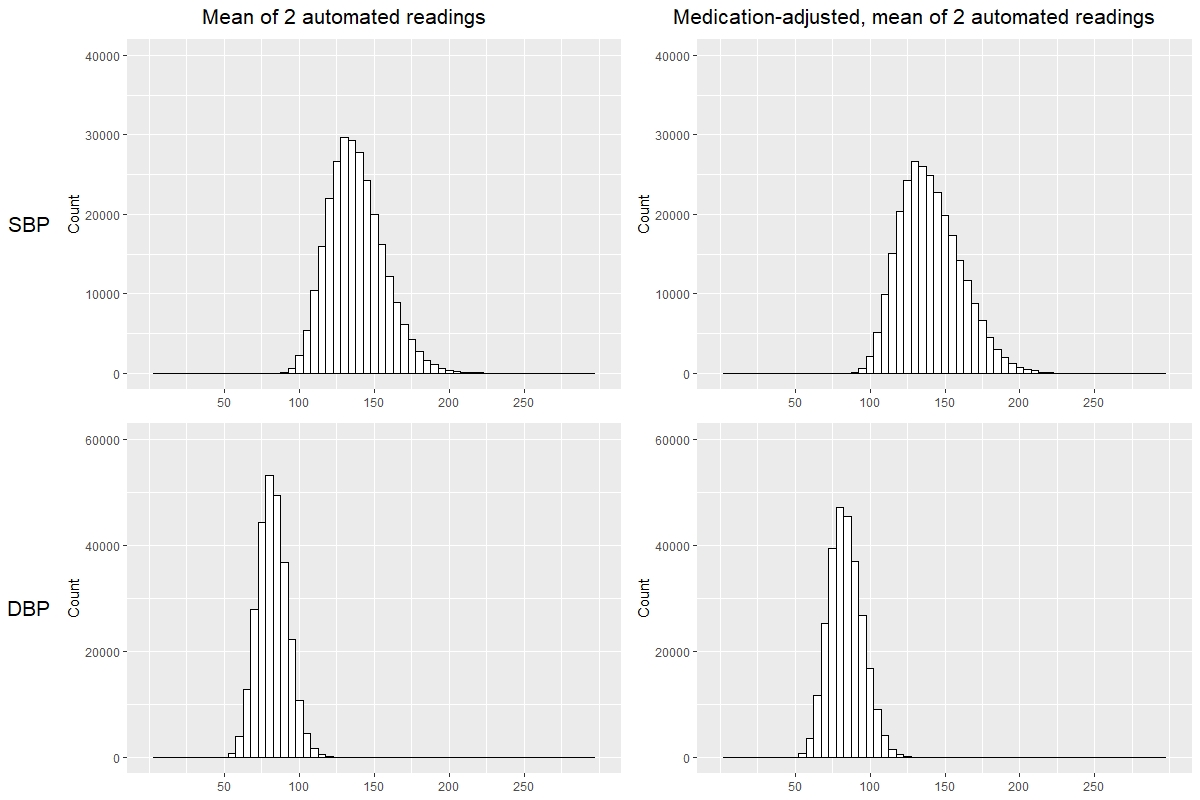
**

**Fig. S2 Distribution of systolic blood pressure (SBP) and diastolic blood pressure (DBP) in 286 390 European participants without metabolomic data from UK Biobank.**

**
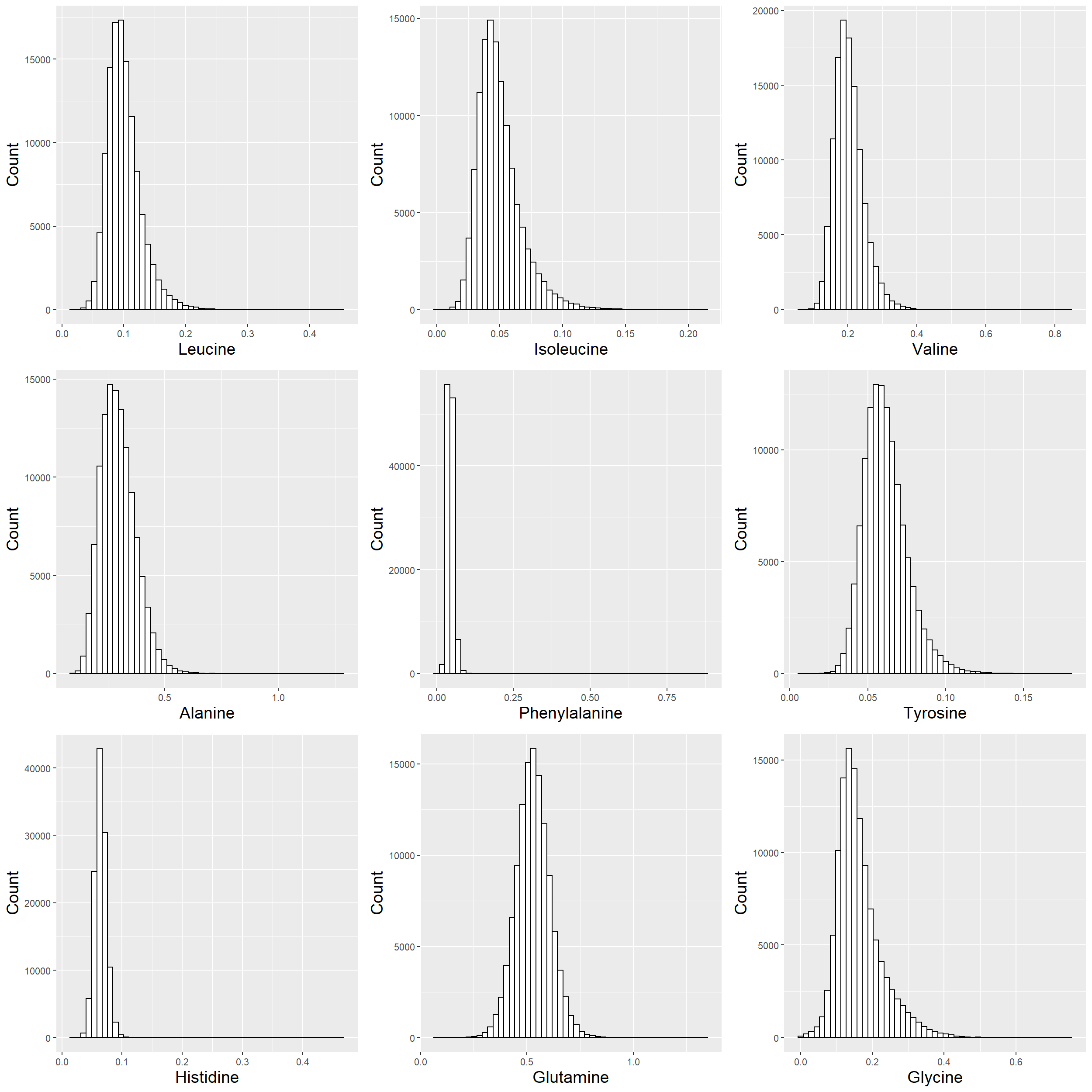
**

**Fig. S3 Distribution of circulating levels of amino acids in 98 317 European participants with metabolomic data from UK Biobank.**





**Fig. S4 Estimated causal effects of circulating levels of amino acids on blood pressure and risk of hypertension.** Causal estimates were obtained using the inverse variance weighted method. Color based on different sets of IV in the analyses (blue, restricted sets of IV as main analyses; dark red, broad sets of IV as secondary analyses).

**

**

**Fig. S5 Scatter plots of SNPs used as instrumental variables for the MR analyses of circulating amino acids with (A)systolic blood pressure, (B) diastolic blood pressure and (C)hypertension.**


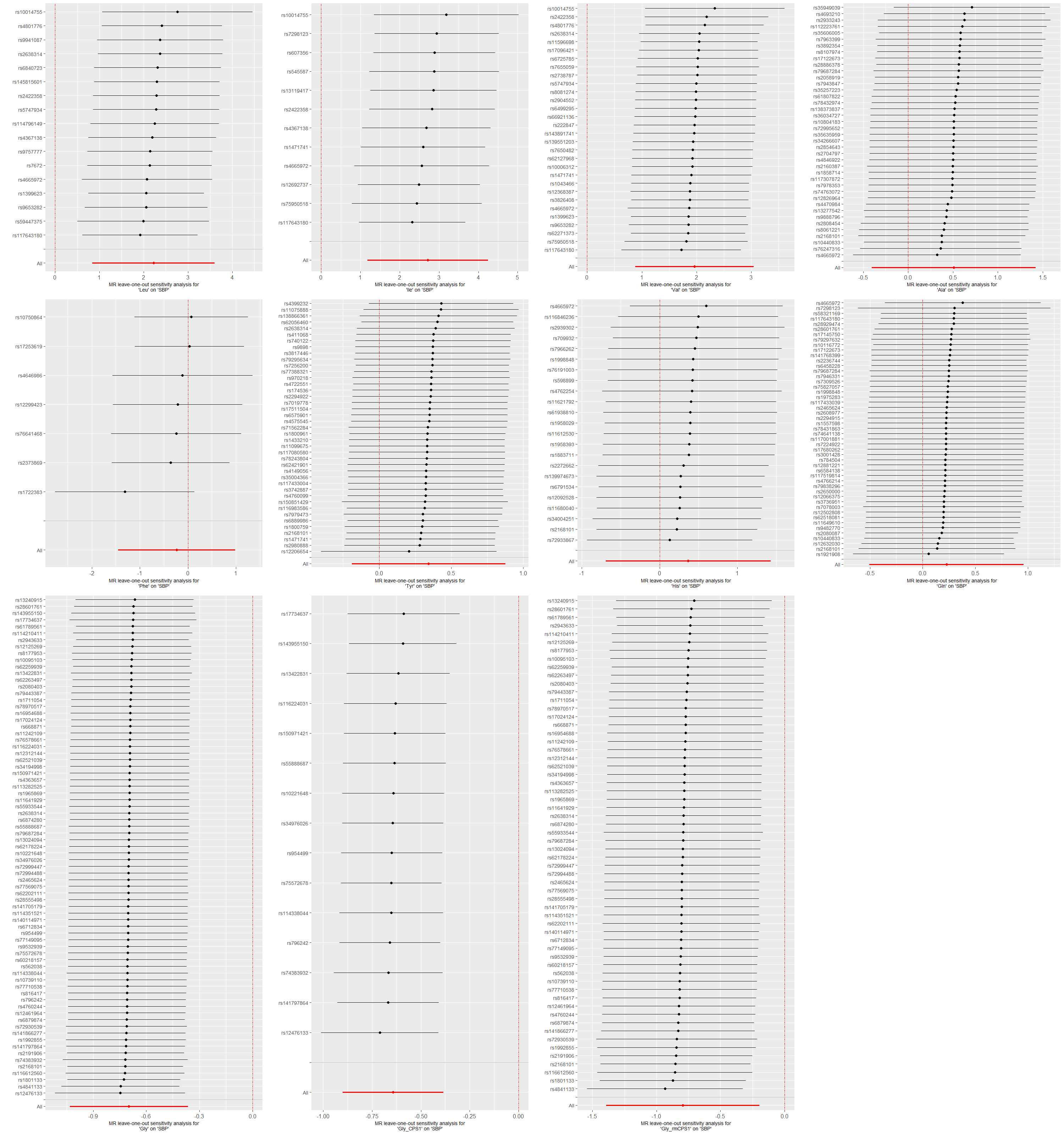


**Fig. S6 Leave-one-out plots to assess if a single SNP is driving the causal effects of circulating amino acids on systolic blood pressure (SBP).**


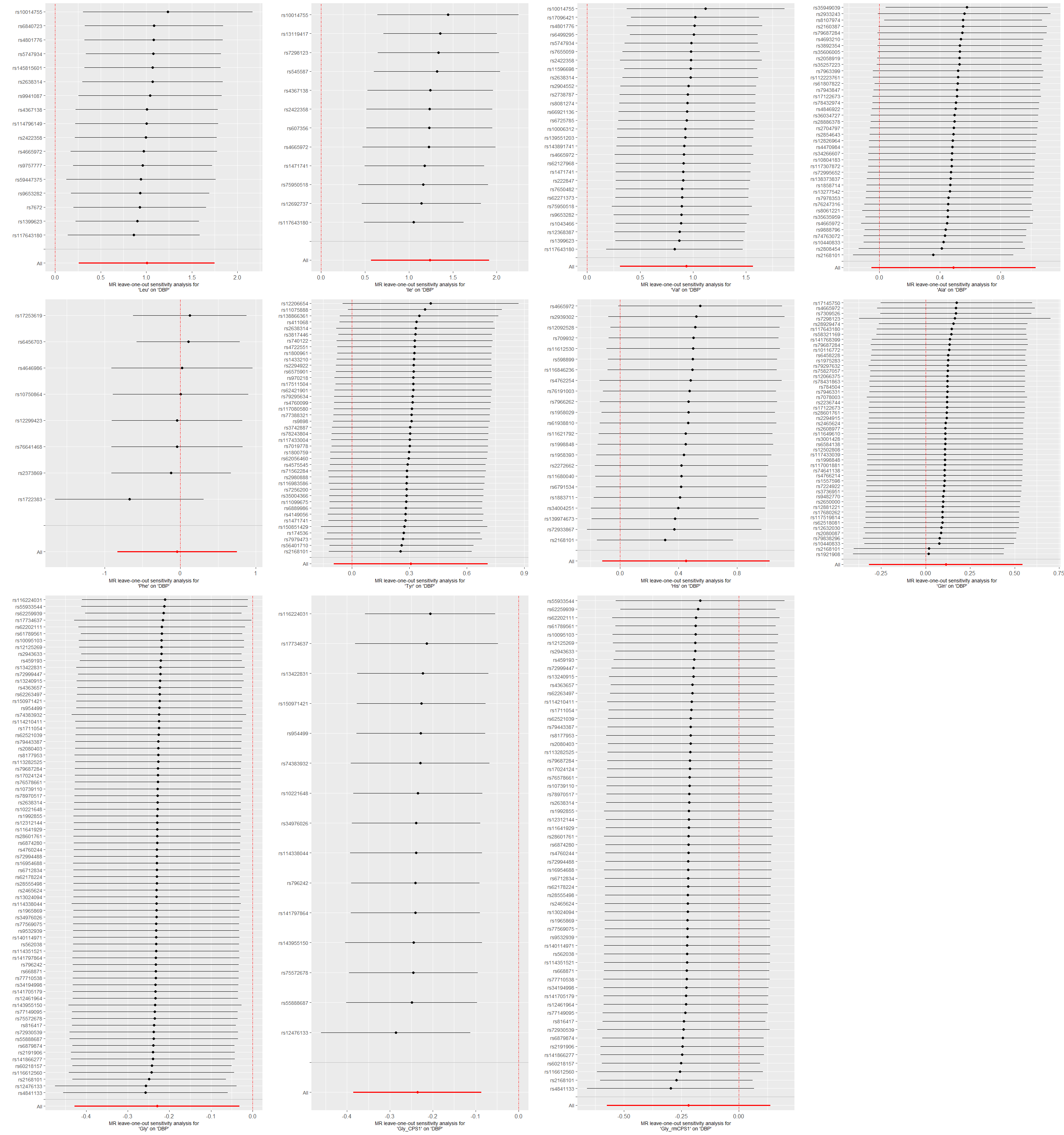


**Fig. S7 Leave-one-out plots to assess if a single SNP is driving the causal effects of circulating amino acids on diastolic blood pressure (DBP).**

**
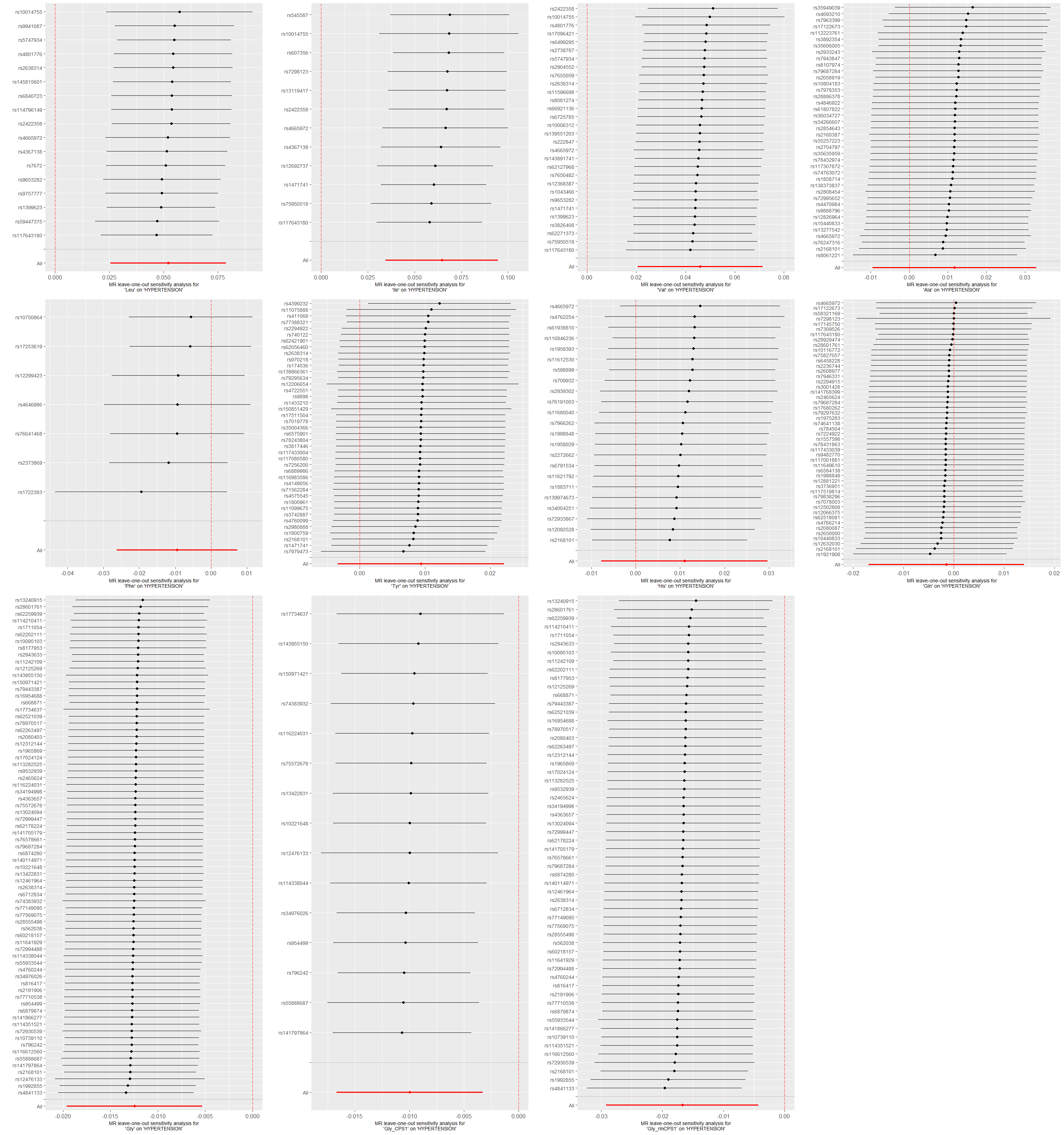
**

**Fig. S8 Leave-one-out plots to assess if a single SNP is driving the causal effects of circulating amino acids on hypertension.**





**Fig. S9 Scatter plots of SNPs used as IVs for the reverse MR analyses of (A)systolic blood pressure, (B) diastolic blood pressure and (C) hypertension with circulating levels of amino acids.**
